# Cultural variation in the SES-gender interaction in student achievement

**DOI:** 10.3389/fpsyg.2023.1120211

**Published:** 2023-09-18

**Authors:** Kimmo Eriksson, Jannika Lindvall

**Affiliations:** ^1^School of Education, Culture and Communication, Mälardalen University, Västerås, Sweden; ^2^Institute for Futures Studies, Stockholm, Sweden

**Keywords:** achievement gap, socioeconomic status, gender differences, international large-scale assessment, cultural variation

## Abstract

**Introduction:**

Is the socioeconomic gap in academic achievement larger among boys than girls? Several scholars have proposed such an interaction between socioeconomic status (SES) and gender. Prior empirical studies have yielded mixed evidence, but they have been conducted almost exclusively in Western countries. Here we propose the hypothesis that the SES-gender interaction is stronger in less gender-equal societies.

**Methods:**

We estimated the SES-gender interaction in 36 countries using data from two international large-scale assessments (PIRLS and TIMSS). The degree of gender equality was measured by the Global Gender Gap Index.

**Results:**

Consistent with the hypothesis, the SES-gender interaction was stronger in societies with less gender equality.

**Discussion:**

Our findings suggest that cultural factors determine how the socioeconomic achievement gap differs between boys and girls.

## Introduction

Insufficient equity in education is recognized as one of the major challenges in school systems around the world (OECD, 2007; Ainscow et al., 2012). Countless studies have documented that students with richer and more well-educated parents tend to perform better in school (White, 1982; Sirin, 2005; Harwell et al., 2017). Although this phenomenon appears to be quite universal, the *size* of the achievement gap between students with high and low socioeconomic status (SES) is not constant. For example, the gap appears to change with time (Chmielewski, 2019) and to be moderated by school factors (Gustafsson et al., 2018). Here we are concerned with another potential moderator of the SES achievement gap: gender.

More than 30 years ago, a Swedish study found that SES gaps in scores achievement tests were larger among boys than girls (Fischbein, 1990). Consistent with this finding, several authors have proposed theoretical arguments predicting larger SES gaps in achievement among boys than girls (Connolly, 2006; Entwisle et al., 2007; Auwarter and Aruguete, 2008; Autor et al., 2019). There is some empirical support for this prediction. In addition to the Swedish study, larger SES achievement gaps among boys than girls have been observed in several studies in several Western countries: Denmark, the United Kingdom, and the United States (Entwisle et al., 2007; Penner and Paret, 2008; Mensah and Kiernan, 2010; Glaesser and Cooper, 2012; Brenøe and Lundberg, 2018; Autor et al., 2019). However, this finding was not replicated in several other studies, similarly conducted in Western countries: New Zealand, Norway, Ireland, the United Kingdom, and the United States (Gibb et al., 2008; Fryer and Levitt, 2010; Strand, 2014; Lenes et al., 2022; McGinnity et al., 2022). Thus, while theorists have predicted larger SES achievement gaps among boys than girls, empirical studies in Western countries have not robustly obtained this finding.

It is well-known that psychological and behavioral findings obtained in Western countries are not necessarily representative of humanity at large (Henrich et al., 2010). Might gender be a stronger moderator of SES achievement gaps in other parts of the world? This question is not answered in prior studies. Indeed, we only found two non-Western studies of how gender moderates the SES achievement gap (Zhu et al., 2018; Liu et al., 2022), and their findings are difficult to interpret because they used SES data reported by students. Student-reported SES data has the drawback that gender differences in SES achievement gaps are conflated with gender differences in data reporting accuracy. Indeed, we have found in ongoing work that boys tend to report SES data less accurately than girls do, causing greater attenuation of SES achievement gaps among boys so that they look narrower than they are (For this reason, we did not include a handful studies that use student-reported SES data in our above review of Western studies: Deslandes et al., 1998; Connolly, 2006; McGraw et al., 2006; Glaesser and Cooper, 2012; Contini et al., 2017).

In short, it is currently unknown whether the Western pattern of results, with no robust gender difference in the size of SES achievement gaps, holds universally or whether culture plays a role. Next, we examine prior theories in order to analyze whether their applicability may differ between cultures.

### Theories about gender moderating SES achievement gaps

The literature describes several mechanisms that could underlie SES achievement gaps and cause them to be larger among boys than girls. For a recent summary of the various mechanisms that have been suggested to underlie SES achievement gaps, see Rözer and van de Werfhorst (2019). Here, we only describe those mechanisms that have been suggested to interact with gender. Our descriptions are brief and only aim to convey the gist of the ideas.

One mechanism that may cause SES achievement gaps is parents’ ability to invest financially in their children’s education (Jerrim and Macmillan, 2015). For several reasons, such as expected returns of investment, parents may on average invest more in the education of their sons than daughters (Alderman and King, 1998). Parents’ ability to invest financially in their children’s education may therefore have a greater impact on investments in boys than in girls, and by extension a greater impact on boys’ than girls’ achievement in school.

Another mechanism is that lower-SES parents may have less time to spend with their children (Jerrim and Macmillan, 2015). It has been suggested that low-SES households are disproportionately female-headed and therefore might spend more time mentoring and interacting with daughters than sons (Autor et al., 2019). If so, the expected consequence would be a greater SES achievement gap among boys than girls.

A third mechanism behind SES achievement gaps is that parents, teachers, and peers tend to have lower expectations for children from more disadvantaged backgrounds (Seginer, 1983; Alvidrez and Weinstein, 1999; Wentzel and Muenks, 2016). Several authors have argued that this will cause wider SES-achievement gaps among boys than girls, because parents’ and teachers’ expectations regarding disadvantaged boys may be especially low, and disruptive peer group norms may be especially prevalent among disadvantaged boys (Connolly, 2006; Entwisle et al., 2007; Auwarter and Aruguete, 2008).

### Could gender equality play a role?

The previous section presented three theoretical arguments for expecting SES-achievement gaps to be larger among boys than girls. Why then is this outcome not robustly observed in studies in Western countries? Here we suggest the possibility that it has to do with Western countries being among the most gender-equal societies in the world (World Economic Forum, 2020). Namely, all the arguments rely on assumptions of boys and girls not being treated equally with respect to parents’ financial investments, parents’ time, and parents’, teachers’, and peers’ expectations. The validity of these assumptions may be weaker in more gender-equal societies and stronger in less gender-equal societies. If this is the case, then measures of SES achievement gaps among boys and girls in societies around the world should reveal a larger gender difference in less gender-equal societies.

### Outline of the present study

The aim of our study is to test the hypothesis that the expected gender difference in the size of SES achievement gaps is more prominent in less gender-equal societies. Our research strategy has two steps: first, to obtain cross-nationally comparable estimates of the SES-gender interaction in numerous countries; second, to examine how these estimates correlate with the levels of gender equality in these countries.

For the first step, international large-scale assessments of student achievement provide an ideal source of data. Many countries around the world participate in these assessments, in which students belonging to a fixed age group take standardized achievement tests. The tests are accompanied by standardized questionnaires that include socioeconomic indicators. From such data we can estimate the SES-gender interaction in each country, and these estimates will be comparable between countries as the students are of the same age group, they take the same test, and their socioeconomic status is appraised in the same way.

For the second step, we require data on how countries differ in their levels of gender equality. Such data are not present in the student achievement assessments. Instead, we use data on gender equality compiled by the World Economic Forum. Combination of data from international large-scale assessments with country data from other sources is a commonly used method to test hypotheses about how educational outcomes vary across societies (e.g., Marks, 2005; Stoet and Geary, 2013; Eriksson et al., 2020).

## Methods

International large-scale assessments of student achievement tend to target either fourth-year students (around age 10) or adolescents (around age 14–15). Our hypothesis is applicable to both age groups. However, the quality of SES data (i.e., parents’ education and occupation) differs between the two age groups. SES data for adolescents is reported by students, and student-reported SES data is not very reliable (Lien et al., 2001; Avvisati, 2020). Moreover, the reliability of student data varies across countries (Jerrim and Micklewright, 2014), and there may well be gender differences in reliability too. To avoid these problems we will instead focus on assessments of fourth-year students, in which SES data are provided by parents. Specifically, we use data from the Progress in International Reading Literacy Study (PIRLS) and the Trends in International Mathematics and Science Study (TIMSS), which are organized by the International Association for the Evaluation of Educational Achievement (IEA). We use data from 36 countries that participated in 2016 PIRLS as well as 2019 TIMSS (the most recent waves of these assessments) and that included the SES measures in the questionnaires to parents. This study was not preregistered.

### Samples

PIRLS and TIMSS use representative samples of students from the participating countries. For details on the sampling strategies, see the official reports (Mullis and Martin, 2015, 2017). Table 1 describes the total sample size per data source.

**Table 1:** Samples sizes and percentages of missing data.

Country	TIMSS				PIRLS			
	Sample	Percentage missing data			Sample	Percentage missing data		
	Size	Gender	Educ.	Occ.	Size	Gender	Educ.	Occ.
Austria	4,464	0	12	22	4,360	0	8	12
Azerbaijan	5,245	2	8	20	5,994	0	19	18
Bahrain	5,762	0	12	29	5,480	0	17	24
Belgium	4,655	0	10	12	5,198	1	13	16
Bulgaria	4,268	0	3	6	4,281	0	3	6
Canada	13,653	9	32	34	18,245	1	19	25
Chile	4,174	1	9	20	4,294	1	12	21
Czech Republic	4,692	4	17	21	5,537	0	8	10
Denmark	3,227	1	42	43	3,508	1	14	10
Finland	4,730	1	12	14	4,896	0	8	10
France	4,186	4	11	16	4,767	1	12	17
Georgia	3,787	6	4	21	5,741	0	7	25
Germany	3,437	13	35	38	3,959	11	28	41
Hungary	4,571	2	9	13	4,623	0	7	10
Iran	6,010	0	3	12	4,385	0	4	9
Ireland	4,582	0	7	12	4,607	0	8	15
Italy	3,741	0	10	11	3,940	0	12	13
Kazakhstan	4,791	0	5	8	4,925	0	9	4
Latvia	4,481	1	8	11	4,157	0	10	12
Lithuania	3,741	3	19	23	4,317	0	17	22
Malta	3,630	0	29	33	3,647	0	23	20
Morocco	7,723	0	16	22	5,489	0	19	29
New Zealand	5,019	1	60	60	5,646	1	52	54
Oman	6,814	0	9	20	9,234	0	11	20
Poland	4,882	0	7	9	4,413	0	3	7
Portugal	4,300	0	7	13	4,642	0	4	10
Qatar	4,933	0	22	33	9,077	0	22	30
Russia	4,022	0	1	5	4,577	0	2	4
Saudi Arabia	5,453	0	11	28	4,741	0	11	25
Singapore	5,986	0	5	8	6,488	0	6	9
Slovakia	4,247	0	5	11	5,451	0	7	10
South Africa	11,891	0	29	41	5,282	2	52	55
Spain	9,555	0	12	18	14,595	0	10	14
Sweden	3,965	1	23	22	4,525	2	20	19
Taiwan	3,765	0	1	4	4,326	0	2	6
United Arab Emir.	25,834	1	54	58	16,471	0	19	24

### Achievement measures

The test scores in the initial waves of PIRLS and TIMSS were standardized to achieve a global mean score of 500 points and a global standard deviation of 100 points; test scores in subsequent waves of these assessments have been calibrated to be comparable to the initial waves. In order to test a wide set of skills, the full tests used in international large-scale assessments are so comprehensive that students cannot take the tests in their entirety. Instead, a rotated test booklet design is used whereby every student only takes a subset of the full test, and a set of five plausible values for the student’s score on the full test is then calculated (Martin et al., 2020).

In PIRLS, there is a single test of reading achievement. In TIMSS, every participant takes both a test in mathematics and a test in science. We use the average of the student’s math and science scores in our analyses in this paper, as theories for the gender difference in SES achievement gaps do not take the specific subject into account.^1^

### Gender and socioeconomic status

Student gender is coded 1 for boy and 0 for girls. We operationalize student SES by two standard measures available in PIRLS and TIMSS: parents’ highest education level and parents’ highest occupation level. Data on parents’ highest education level are given on a five-step scale: primary school or no school (coded 1), lower secondary (2), upper secondary (3), post-secondary but not university (4), university or higher (5); data on parents’ highest occupation level are given on a six-step scale: never worked for pay (1), general laborer (2), skilled worker (3), clerical (4), small business owner (5), and professional (6). See Martin et al. (2020).

In each country, we mean center the measures of gender, parents’ highest education level, and parents’ highest occupation level. That is, from each variable we subtract its mean value in the country.

### Country levels of gender equality and development

We measure the level of gender equality in a country by its Global Gender Gap Index (GGGI). This is a number between 0 and 1, where higher values mean more equality. The GGGI is based on gender gaps in the domains of economics, politics, education, and health; scores for 2019 were obtained from the World Economic Forum (2020).^2^

As a control variable we use the level of development in a country, measured by the Human Development Index (HDI). This is a number between 0 and 1, where higher values mean higher development. The HDI is based on indicators of the levels of health, education, and standard of living in the country; score for 2019 were obtained from the United Nations Development Programme (2020).^3^

### Analysis

The first step in the analysis is the estimation of the SES-gender interaction in each country. We use two different operationalizations of SES (education and occupation) and analyze two different assessments (PIRLS and TIMSS). For each country we thus estimate the SES-gender interaction four times. Estimation is done by multiple linear regression of test scores on the SES measure, gender, and the interaction term (SES × gender). The coefficient of the latter term is our estimate of the SES-gender interaction. To perform the multiple linear regression, we use SPSS syntax created by the IDB Analyzer, a software tool provided by IAE.^4^ The IDB Analyzer handles analysis of plausible values by computing results for each plausible value and combining these estimates using Rubin-Shaffer rules (Rutkowski et al., 2010).

The second step in the analysis is examination of how the SES-gender interaction varies with the level of gender equality. In this step we perform country-level correlations between gender equality and the estimates of the SES-gender interaction obtained in step 1, both with and without controlling for the level of development. As results are similar for the four different estimates of the SES-gender interaction, we create a single index for the SES-gender interaction by taking the average of the four estimates (Cronbach’s α = 0.63). We use this index to provide a single graphical illustration of the correlation between gender equality and the size of the SES-gender interaction. We also use the index in a hierarchical regression to demonstrate that the level of gender equality influences the SES-gender interaction above and beyond the level of development.

### Missing data

The percentages of missing data on gender, parents’ highest education, and parents’ highest occupation in each country are given in Table 1. These percentages vary widely. In some countries, like Bulgaria or Taiwan, almost no data is missing. In New Zealand, by contrast, most SES data are missing. Missing data is unlikely to be a problem for this study, however. In order for missing data to bias estimates of relation between the SES-gender interaction and gender equality, they must show a very particular pattern (i.e., data need to be especially likely to be missing for students who have a specific gender AND a specific socioeconomic status AND a specific level of achievement AND live in a country with a specific level of gender equality). Moreover, we checked that country percentages of missing data are not significantly correlated with country estimates of the SES-gender interaction or with country levels of gender equality (Supplementary Table S1).

**Data availability statement.** The raw data used in step 1 are publicly available at IEA’s website (iea.nl). All country measures used in step 2 are available at OSF (https://osf.io/exkcm/?view_only=fc3a613c2a7c4d91b8ff9bc29ce7c7fd).

## Results

Descriptive statistics of the country-level estimates of the SES-gender interaction and, for completeness, the main effects of SES and gender are presented in Table 2. While the main effects are not our focus of interest, note that there is a robust positive effect of SES on achievement, whereas the gender effect varies across assessments between a consistent disadvantage for boys in PIRLS (i.e., in reading) and, on average, a slight advantage for boys in TIMSS (i.e., in mathematics and science). The focus of our interest is the SES-gender interaction, which is on average slightly positive. For example, when SES is measured by parents’ highest education the SES-gender interaction in PIRLS has a mean value of 2.5, while the mean value of the main effect of SES is about 10 times larger. This means that the effect of parents’ education on reading achievement is roughly 10% larger among boys than girls in the average country.

**Table 2:** Descriptive statistics of country estimates of the main effects of SES and gender, and the SES-gender interaction, on student achievement in international large-scale assessments.

SES measure	Assessment	SES	Gender	SES × Gender
Education	PIRLS	24.6 (7.6)	−18.5 (13.3)	2.5 (4.4)
	TIMSS	25.4 (7.3)	1.6 (10.9)	1.4 (3.4)
Occupation	PIRLS	16.0 (5.6)	−18.1 (12.3)	0.8 (3.3)
	TIMSS	16.4 (5.9)	2.0 (10.5)	1.1 (2.4)

*N* = 36 countries. Entries are mean values with standard deviations in parentheses.

**Figure 1:**
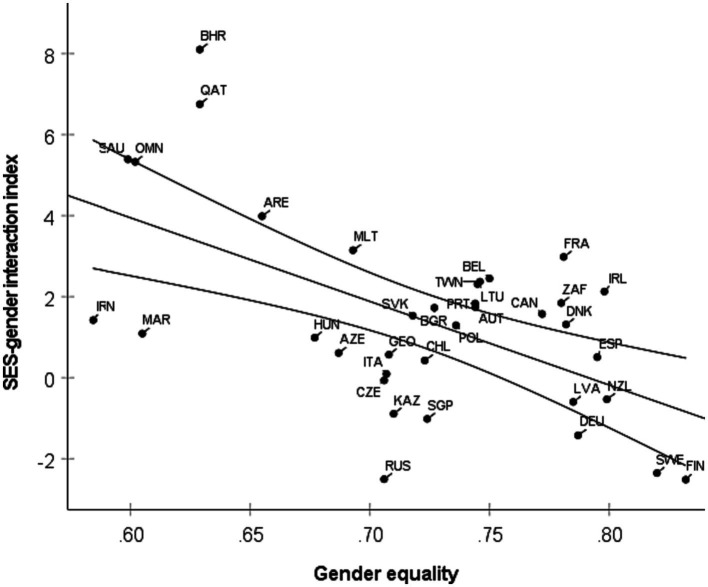
A scatterplot of the SES-gender interaction index against a measure of the level of gender equality in 36 countries. The SES-gender interaction index is the average of the four estimates of the difference between boys and girls in the effects of parents’ education and occupation on student achievement in TIMSS and PIRLS. Gender equality is measured by the Global Gender Gap Index. Countries are referred to by their ISO 3-letter country code (Supplementary Table S2). The negative slope of the regression line (with 95% confidence interval) means that the SES-gender interaction is greater in less gender-equal countries. For example, the dot marked SAU refers to Saudi Arabia, a country with a relatively low level of gender equality (0.60) and a relatively large SES-gender interaction index (5.4). The latter value signifies that in Saudi Arabia, the average effect on achievement scores in TIMSS and PISA of having one-unit higher scores on parents’ education and occupation was 5.4 points higher among boys (12.9) than among girls (7.5).

However, there is considerable variation across countries; for example, the effect of parents’ education on reading achievement is almost 300% larger among boys than girls in Saudi Arabia, which is among the least gender-equal countries in this study according to the GGGI measure (Supplementary Table S2). Table 3 reports how each of the estimated effects correlated with the level of gender equality of countries. In support of the hypothesis of the current study, gender equality is negatively correlated with SES-gender interactions (whether or not we control for the development level of countries). In other words, it is especially in less gender-equal countries that the effect of SES on achievement is larger among boys than girls. The scatterplot in Figure 1 illustrates this finding by showing how the most gender-unequal countries tend to have the largest SES-gender interaction, that is, the greatest gender difference in the SES effect. The hierarchical linear regression in Table 4 demonstrates that the level of gender equality in countries explains their SES-gender interaction above and beyond the level of development.

**Table 3:** Correlations with country levels of gender equality for the country estimates of main and interaction effects of SES and gender on student achievement.

SES measure	Assessment	Correlation between GGGI and the estimated effect of		
		SES	Gender	SES × Gender
Education	PIRLS	−0.18	0.64^***^	−0.56^***^ (−0.54^***^)
	TIMSS	0.07	0.39^*^	−0.29^**^ (−0.40^*^)
Occupation	PIRLS	0.12	0.63^***^	−0.56^***^ (−0.56^***^)
	TIMSS	0.31^†^	0.38^*^	−0.05 (−0.16)

^*^p < 0.05; ^**^p < 0.1; ^***^p < 0.001. *N* = 36 countries. Entries are Pearson correlations. GGGI is the Global Gender Gap Index. Within parentheses are partial correlations controlling for the Human Development Index.

**Table 4:** Results of hierarchical linear regression of the SES-gender interaction index.

Variable	Model 1	Model 2
HDI	−0.08	0.30
GGGI		−0.72^***^
*R* ^2^	0.01	0.39
*R*^2^ change		0.38

^***^p < 0.001; ^*^p < 0.05; ^**^p < 0.1. Entries are standardized coefficients. HDI is the Human Development Index. GGGI is the Global Gender Gap Index. *R*^2^ is the proportion of variance explained by the model.

## Discussion

There are several theoretical reasons to expect SES achievement gaps to be larger among boys than girls, but prior studies have not found a robust gender difference in the size of SES achievement gaps. As prior studies were almost exclusively conducted in Western countries, we considered the possibility that different results may be obtained in other parts of the world.

From two international large-scale assessments we obtained data on SES, gender, and achievement that allowed us to estimate the SES-gender interaction in 36 countries across the world. Consistent with prior studies, estimates of the SES-gender interaction in Western countries did not have a consistent sign. However, in certain non-Western countries—like Bahrain, Qatar, Oman, Saudi Arabia, and the United Arab Emirates—the effect of SES on achievement was considerably larger among boys than girls. Moreover, these countries all have low levels of gender equality (as measured by the World Economic Forum). The moderating effect of gender equality was found regardless of whether SES was operationalized by parents’ education or parents’ occupation, and whether achievement was measured in the reading domain (PIRLS) or in the math-science domain (TIMSS).

Our interpretation of these results is that SES achievement gaps to some extent depend on how children are treated. In more gender-equal societies, boys and girls are treated more equally, which would explain why there are little gender differences in the SES achievement gaps in these societies. Unequal treatment of boys and girls may manifest in, say, differences in parental investments in boys’ and girls’ education, or differences in parental expectations on boys and girls. Prior theories of SES-gender interactions in student achievement assume that such differences in how boys and girls are treated may interact with socioeconomic differences (Connolly, 2006; Entwisle et al., 2007; Auwarter and Aruguete, 2008; Autor et al., 2019). The novelty in the current study lies in that we consider how gender differences in treatment may vary with the level of gender equality of the society.

When estimating the SES-gender interaction, we also obtained estimates of the main effects of SES and gender on achievement. Country variation in SES achievement gaps was not related to gender equality. Other studies have examined other country factors that may explain why SES achievement gaps vary in size (e.g., Marks, 2005; Bodovski et al., 2017). By contrast, there was a strong correlation in our data between the main effect of gender on achievement and gender equality; boys achieve better, relative to girls, in more gender-equal societies. For a more detailed examination of this phenomenon, see Eriksson et al. (2020).

A limitation of our study is that it only covers 36 countries. The world is large and it would be interesting to see how SES achievement gaps differ between boys and girls in, say, African countries. Another limitation is that there may be omitted variables that confound the effect of gender equality. In the current study we controlled for the overall development level of the countries, but there may be other important variables that we did not control for. To examine whether the level of equality in the treatment of boys and girls has a causal effect on gender differences in SES achievement gaps, intervention studies could be conducted. It would be valuable to know whether SES achievement gap among boys can be reduced in size through measures that address specific ways in which boys and girls are treated differently. Another possibility is to study how the SES-gender interaction varies across ethnicities in the same country (Strand, 2014), to see whether it is related to ethnic differences in the level of gender egalitarianism.

Gender equality has increased globally for many decades (Inglehart and Norris, 2003). If there is a causal connection between low gender equality and the size of the SES-gender interaction in student achievement, we should therefore expect that SES-gender interaction has decreased over time. Thus, our findings motivate future longitudinal studies of the same topic.

In conclusion, this study has documented that socioeconomic achievement gaps are often larger among boys and girls, and especially in non-Western societies with high levels of gender inequality.

## Data availability statement

The raw data used in step 1 are publicly available at IEA’s website (iea.nl). All country measures used in step 2 are available at OSF (https://osf.io/exkcm/).

## Ethics statement

Ethical approval was not required for the study involving humans in accordance with the local legislation and institutional requirements. Written informed consent to participate in this study was not required from the participants or the participants’ legal guardians/next of kin in accordance with the national legislation and the institutional requirements.

## Author contributions

KE conceived of the study, performed the analysis, and wrote the paper. JL conducted the survey of the literature and provided critical feedback on the manuscript. All authors contributed to the article and approved the submitted version.

## Funding

This work was supported by the Swedish Research Council under Grant 2014–2008.

## Conflict of interest

The authors declare that the research was conducted in the absence of any commercial or financial relationships that could be construed as a potential conflict of interest.

## Publisher’s note

All claims expressed in this article are solely those of the authors and do not necessarily represent those of their affiliated organizations, or those of the publisher, the editors and the reviewers. Any product that may be evaluated in this article, or claim that may be made by its manufacturer, is not guaranteed or endorsed by the publisher.

## Supplementary material

The Supplementary material for this article can be found online at: https://www.frontiersin.org/articles/10.3389/fpsyg.2023.1120211/full#supplementary-material

## Footnotes

^1^In supplementary analyses, we estimated the SES-gender interaction separately for math and science scores. The results were virtually identical to the ones we report for the average scores; country-level correlations between the estimates of the SES-gender interaction that we report and those we obtain from only math scores or only science scores are almost perfect, *r* = 0.97. Hence, separating subjects do not add anything to our understanding of how the SES-gender interaction varies across countries.

^2^The score for Taiwan was obtained from the report “2021 Gender at a glance in R.O.C. (Taiwan),” https://www.boca.gov.tw/dl-2644-65dec9e6cec54e1da10f3690bf1a65ec.html (Accessed January 31, 2023).

^3^The score for Taiwan was obtained from the Subnational Human Development Database (Smits and Permanyer, 2019).

^4^ https://www.iea.nl/data-tools/tools
