# Supplementary material for: Cultural variation in the SES-gender interaction in student achievement
Source: Front Psychol. 2023 Sep 18;14:1120211. doi: 10.3389/fpsyg.2023.1120211 (PMC10547582; doi:10.3389/fpsyg.2023.1120211)
Supplement: Supplementary file 1 [file Table_1.DOCX]

**Supplementary Table 1.** Correlations between percentages of missing data and estimates of the SES-gender interaction.

|  |  | Correlation between estimates of the SES-gender interaction and percentages of missing data on | | |
| --- | --- | --- | --- | --- |
| SES variable | Data source | gender | education | occupation |
| Education | TIMSS | -.02 | .13 | .20 |
| Occupation | TIMSS | -.14 | .11 | .12 |
| Education | PIRLS | -.25 | .13 | .30 |
| Occupation | PIRLS | -.29 | .02 | .30 |

Note. No correlation is statistically significant at *p* < .05.
