# Supplementary material for: Cultural variation in the SES-gender interaction in student achievement
Source: Front Psychol. 2023 Sep 18;14:1120211. doi: 10.3389/fpsyg.2023.1120211 (PMC10547582; doi:10.3389/fpsyg.2023.1120211)
Supplement: Supplementary file 2 [file Table_2.DOCX]

**Supplementary Table 2.** Estimates of the effect of SES on achievement among boys and girls.

|  |  | |  | TIMSS | | | | PIRLS | | | |
| --- | --- | --- | --- | --- | --- | --- | --- | --- | --- | --- | --- |
|  |  | |  | education | | occupation | | education | | occupation | |
| Country | | ISO-3 | GGGI | boys | girls | boys | girls | boys | girls | boys | girls |
| Austria | | AUT | .74 | 29.3 | 25.2 | 27.2 | 25.6 | 20.6 | 17.5 | 16.6 | 18.5 |
| Azerbaijan | | AZE | .69 | 16.3 | 14.7 | 22.7 | 21.4 | 7.5 | 7.6 | 10.1 | 10.5 |
| Bahrain | | BHR | .63 | 20.6 | 15.1 | 37.8 | 23.5 | 9.6 | 7.4 | 22.0 | 11.6 |
| Belgium | | BEL | .75 | 23.8 | 18.7 | 17.3 | 16.5 | 19.0 | 16.5 | 13.2 | 11.8 |
| Bulgaria | | BGR | .73 | 41.4 | 38.2 | 36.0 | 35.7 | 32.0 | 28.8 | 27.5 | 27.4 |
| Canada | | CAN | .77 | 28.1 | 24.3 | 25.7 | 25.6 | 13.5 | 13.2 | 15.5 | 13.5 |
| Chile | | CHL | .72 | 28.6 | 23.7 | 26.3 | 26.8 | 16.0 | 13.8 | 12.8 | 17.7 |
| Czech Repub. | | CZE | .71 | 25.0 | 24.9 | 24.8 | 22.2 | 17.9 | 19.2 | 15.3 | 17.0 |
| Denmark | | DNK | .78 | 23.5 | 18.0 | 18.5 | 22.2 | 19.5 | 11.7 | 15.5 | 19.9 |
| Finland | | FIN | .83 | 15.6 | 22.8 | 21.5 | 19.9 | 13.5 | 16.0 | 13.0 | 14.8 |
| France | | FRA | .78 | 34.5 | 33.4 | 23.3 | 18.6 | 23.1 | 21.5 | 16.7 | 12.4 |
| Georgia | | GEO | .71 | 18.5 | 18.7 | 28.9 | 24.7 | 4.7 | 6.4 | 7.7 | 7.8 |
| Germany | | DEU | .79 | 21.8 | 21.8 | 24.0 | 26.7 | 19.0 | 18.3 | 16.9 | 20.6 |
| Hungary | | HUN | .68 | 36.1 | 33.8 | 33.5 | 33.8 | 25.5 | 23.1 | 22.9 | 23.3 |
| Iran | | IRN | .58 | 26.5 | 31.5 | 32.0 | 23.5 | 13.9 | 17.4 | 15.1 | 9.3 |
| Ireland | | IRL | .80 | 24.8 | 25.4 | 25.8 | 21.3 | 15.9 | 14.9 | 17.3 | 13.7 |
| Italy | | ITA | .71 | 16.6 | 16.2 | 19.4 | 20.1 | 11.2 | 11.1 | 14.8 | 14.2 |
| Kazakhstan | | KAZ | .71 | 12.4 | 13.3 | 9.3 | 12.5 | 6.8 | 5.7 | 4.7 | 5.3 |
| Latvia | | LVA | .79 | 20.3 | 24.6 | 22.0 | 19.5 | 11.7 | 11.1 | 12.7 | 13.9 |
| Lithuania | | LTU | .75 | 41.6 | 35.8 | 31.0 | 29.4 | 21.9 | 18.8 | 16.7 | 18.0 |
| Malta | | MLT | .69 | 26.1 | 22.1 | 9.0 | 8.8 | 20.6 | 15.3 | 10.0 | 6.9 |
| Morocco | | MAR | .61 | 23.7 | 21.9 | 25.9 | 24.1 | 11.0 | 10.7 | 17.8 | 17.2 |
| New Zealand | | NZL | .80 | 30.8 | 32.7 | 26.8 | 24.4 | 24.6 | 26.5 | 19.5 | 20.3 |
| Oman | | OMN | .60 | 29.9 | 23.4 | 30.9 | 24.5 | 16.1 | 11.8 | 17.1 | 13.0 |
| Poland | | POL | .74 | 27.5 | 25.4 | 20.3 | 20.5 | 20.6 | 16.1 | 14.9 | 16.2 |
| Portugal | | PRT | .74 | 18.0 | 17.8 | 16.2 | 12.9 | 17.9 | 16.9 | 14.7 | 11.9 |
| Qatar | | QAT | .63 | 37.3 | 28.8 | 43.4 | 33.7 | 17.0 | 15.4 | 23.7 | 16.5 |
| Russia | | RUS | .71 | 19.2 | 22.8 | 25.5 | 27.2 | 8.7 | 11.6 | 12.5 | 14.2 |
| Saudi Arabia | | SAU | .60 | 16.1 | 15.1 | 16.9 | 4.3 | 9.3 | 7.2 | 9.4 | 3.5 |
| Singapore | | SGP | .72 | 33.4 | 33.0 | 30.9 | 31.7 | 23.0 | 25.3 | 23.2 | 24.5 |
| Slovakia | | SVK | .72 | 34.7 | 31.8 | 36.4 | 35.1 | 25.6 | 24.4 | 29.7 | 29.1 |
| South Africa | | ZAF | .78 | 41.5 | 43.4 | 39.6 | 32.3 | 24.7 | 24.6 | 27.0 | 25.1 |
| Spain | | ESP | .80 | 16.7 | 16.3 | 13.7 | 13.9 | 15.4 | 14.2 | 12.8 | 12.3 |
| Sweden | | SWE | .82 | 25.4 | 24.9 | 21.1 | 25.7 | 16.9 | 18.4 | 14.7 | 18.4 |
| Taiwan | | TWN | .75 | 23.6 | 22.3 | 22.2 | 18.8 | 17.3 | 14.9 | 14.3 | 11.8 |
| United Arab Emirates | | ARE | .66 | 28.2 | 26.3 | 42.7 | 32.3 | 18.4 | 17.3 | 22.4 | 19.8 |

Note. The SES effect among boys (girls) is calculated as the main effect of SES plus (minus) half the SES-gender interaction effect.
